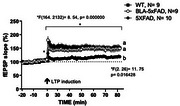# Amygdala stimulation promotes memory recovery and synaptic plasticity in Alzheimer's disease genetic animal model 5XFAD

**DOI:** 10.1002/alz70855_096825

**Published:** 2025-12-23

**Authors:** Daymara Mercerón, Felipe Conejera, Luis Tabilo, Paulina Puebla, William Almaguer, Alvaro Ardiles

**Affiliations:** ^1^ Medicine Faculty, Universidad de Valparaíso, Valparaíso, Valparaíso, Chile; ^2^ Sciences Faculty, Universidad de Valparaíso, Valparaíso, Valparaíso, Chile; ^3^ Biology Faculty, Universidad de Valparaiso, Valparaíso, Valparaíso, Chile; ^4^ International Center for Neurological Restoration, Havana, Havana, Cuba

## Abstract

**Background:**

Alzheimer´s disease is the most common disease in the worldwide and despite the great efforts made at present cure is lacking. In this work we study whether the basolateral region of the amygdala (BLA) stimulation, a brain region known as for its role in emotions and motivations, memory and learning formation, and which is capable of broadly promoting neural plasticity mechanisms, is capable of promote the recovery of spatial memory in the genetic model of Alzheimer's disease known as 5XFAD.

**Method:**

To test this hypothesis, we used 12‐13‐month‐old mice 5XFAD. A bipolar stimulation electrode was attached bilaterally in the BLA through stereotactic surgery and 15 min after behavioral training the BLA was stimulated consisted in 3 trains of 15 pulses at 200 Hz. A week later the animals were trained in the Morris Water Maze (MWM) for 5 days and 15 min after the end tasks BLA was stimulated. Additionally, in order to evaluate if this proceeding produces a general memory recovery we evaluated the animals in other two behavioral tasks, Novel Object Recognition Test (NORT) and Place Recognition Test (PRT). Finally, we search if the combination of MWM plus 15 min after BLA stimulation improved Long‐Term Synaptic Potentiation (LTP), cellular model for learning and memory, which is affected in 5XFAD.

**Result:**

We showed that MWM plus 15 min after BLA stimulation treatment for four days produced a memory recovery in 5XFAD mice, and more important in NORT and PRT too, suggesting is not just facilitation of particular memory trace if not a general recovery of skill learning and memory. Furthermore, MWM plus BLA stimulation treatment also produced an improvement in LTP, especially in its maintenance, since LTP in 5XFAD mice decay rapidly.

**Conclusion:**

Combination MWM plus BLA stimulation is able to induce plasticity related mechanisms involved in learning and memory but also in functional recovery, which produces skill‐memory recovery in the 5XFAD mice. This result opens a path in the search for neurological restoration based on the plasticity of memory loss in Alzheimer's disease.